# Evolution of mucosal vasculature after radiotherapy of T1 vocal cord cancer: a pilot study

**DOI:** 10.1007/s00405-022-07680-5

**Published:** 2022-10-05

**Authors:** Peter Kántor, Lucia Staníková, Jakub Lubojacký, Michaela Masárová, Karol Zeleník, Pavel Komínek

**Affiliations:** 1 Department of Otorhinolaryngology and Head and Neck Surgery, University Hospital Ostrava, 17. listopadu 1790/5, 70800 Ostrava, Czech Republic; 2 Department of Craniofacial Surgery, Faculty of Medicine, University of Ostrava, Syllabova 19, 70300 Ostrava, Czech Republic

**Keywords:** Larynx, Narrow band imaging (NBI), Radiotherapy, Glottic cancer, Endoscopy, Intrapapillary capillary loops

## Abstract

**Purpose:**

Narrow-band imaging is the state of the art in the diagnosis of mucosal lesions of the vocal cords. It is also used in the follow-up of patients after surgical therapy. Unfortunately, if a patient has received radiotherapy the follow-up is much more difficult. Radiation induces inflammatory changes in the mucosa, which lead to changes in the vascular architecture and thus affect the results of the examination. The dynamics and time dependence of vascular changes after radiotherapy have not yet been described. The purpose of this study is to describe the evolution of the vascular pattern in vocal cords after primary radiotherapy for glottic cancer.

**Methods:**

This was a retrospective cohort study. Each patient underwent NBI videolaryngoscopy and was followed every 3 months.

**Results:**

The tumor-related mucosal changes diminished at 3 months after radiotherapy. Afterward, growth of new longitudinal vasculature was observed and significantly slowed after 9 months. No perpendicular vasculature or tumor recurrence was observed during the course of the study.

**Conclusions:**

According to our data, we can conclude that post-radiation mucosal vasculature changes are only longitudinal.

## Introduction

Narrow-band imaging (Olympus Corporation, Tokyo, Japan) was first used in clinical practice in 2005 [1]. Since then, it has become state of the art and an integral part of in-office diagnosis of laryngeal lesions. It uses the enhancement of mucosal intrapapillary capillary loops (IPCLs) with narrow-band light at two wavelengths, 415 and 514 nm [1]. These specific wavelengths allow us to enhance the visibility of the IPCLs in the laryngeal mucosa, which form a specific pattern. Based on the changes in the architecture of the IPCL, the examiner can determine the biological type (benign, premalignant, malignant) of lesion with a high degree of probability, so that some authors even speak of a pre-histological diagnosis. Moreover, the examination provides invaluable information during procedures under general anesthesia and helps the examiner take targeted biopsies and identify the borders of the lesion.

NBI endoscopy is also commonly used in the follow-up of patients after treatment for laryngeal lesions, such as malignant tumors. In patients after treatment for laryngeal tumors, NBI is an invaluable tool for detecting possible tumor recurrence [2]. Interestingly, the usefulness of NBI in the follow-up of patients after primary radiotherapy has not yet been widely studied. The way irradiation affects the vascular pattern and the time dependence of the changes in the IPCLs are not known, and the difficulty of interpreting the results is widely mentioned by other authors [3–5]. The aim of this study was to describe the changes in IPCLs and their dynamics and time dependence after primary radiotherapy for glottic carcinoma. To the authors' best knowledge, no other study on this topic has been published yet.

## Materials and methods

All procedures performed in the study conformed to the ethical standards of the Institutional Research Committee and the 1964 Declaration of Helsinki and its subsequent amendments, or comparable ethical standards. The study protocol was approved by the Ethics Committee of University Hospital Ostrava.

We retrospectively analyzed a cohort of patients treated for T1 glottic carcinoma in the years 2015–2021.

All patients were initially examined in the office. After local anesthesia with 10% lidocaine, a transnasal videolaryngoscopy including NBI was performed. The vascular pattern of the laryngeal lesion was described in writing and a video recording was made.

Next, patients with suspicious lesions were put under general anesthesia and the suspicious lesion was biopsied. The sample was fixed in formalin solution and sent for histopathological examination. In case a malignant tumor was verified, the patient was either treated surgically by appropriate cordectomy or by primary radiotherapy. The decision was done in cooperation with a radiation oncologist and was made on the basis of tumor characteristics and each patient’s preference.

Patient with T1 laryngeal cancer treated primary with radiation therapy were selected for the purpose of our study. Exclusion criteria were tumor stage other than T1, recurrent tumors, indication for primary surgical therapy, and incomplete patient documentation.

After completion of radiotherapy, patients were examined 3, 6, 9, and 12 months after radiotherapy by use of a transnasal videolaryngoscopy with NBI under local anesthesia. A video record was made of each examination, and the vascular changes were recorded descriptively. An overview of the patients and their findings can be found in Table 1.

**Table 1:** Overview of findings and patients in the study

Patient no.	Sex	Age	Histology	RT dose/cycles	Before RT	Mucosal vasculature			
						3 months after RT	6 months after RT	9 months after RT	12 months after RT
1	M	71	SCC	55 Gy/20	L	LV, thin, hair-like	LV, ectatic, increased number	LV, ectatic, increased number, layered	No progression
2	M	50	CIS	55 Gy/20	L, PV	LV, increased number	LV, increased number, ectatic	LV, ectatic, increased number, layered	Minimal progression
3	M	70	SCC	55 Gy/20	L, PV	LV, thin, hair-like	LV, more evident, arborizing	LV, ectatic, arborizing, more evident	Minimal progression
4	M	67	SCC	55 Gy/20	L, PV	LV, thin, hair-like, areas obstructed with white mucosa	LV, more evident	LV, ectatic	No progression
5	M	62	SCC	55 Gy/20	L	LV, thin, hair-like, increased number	LV, ectatic	LV, ectatic, increased number	No progression
6	M	67	SCC	55 Gy/20	L, PV	LV, thin, hair-like, increased number	LV, ectatic, layering	LV, ectatic, increasing number, arborization, areas obstructed with white mucosa	Minimal progression
7	F	65	SCC	55 Gy/20	PV, exophytic tumor	LV, thin, hair-like	LV, ectatic, areas obstructed with white mucosa	LV, ectatic, more evident, white mucosa denser	Minimal progression

*RT* radiotherapy, *M* male, *F* female, *SCC* spinocellular carcinoma, *CIS* carcinoma in situ, *Gy* gray, *L* leukoplakia, *LV* longitudinal vasculature, *PV* perpendicular vasculature

## Results

A total of 50 patients were enrolled in the study. After the application of exclusion criteria, the final cohort consisted of 7 patients. There were 35 patients excluded from the study, because they were primarily treated surgically, and 8 patients were excluded, because they were lost during follow-up or because of incomplete documentation.

Although the sample included only seven patients, it was highly consistent. All patients underwent radiotherapy for either glottic spinocellular carcinoma (six patients) or carcinoma in situ (one patient). The radiation dose was 55 Gy in all patients, and radiation was administered in 20 doses of 2.75 Gy each.

Before radiotherapy, the most common mucosal changes were leukoplakia (6/7 patients). The most common IPCL changes were perpendicular vasculature (according to the ELS classification) or Ni IV, Va–c (according to the Ni et al. classification), which were observed in 5/7 patients [6, 7]. No perpendicular vasculature was observed in 2/7 patients, which was due to the “umbrella effect” of leukoplakia.

At 3 months after radiotherapy, the perpendicular vasculature had diminished and we observed only longitudinal vascularization, which was described in 7/7 patients. The IPCLs were thin and hair-like in all of the patients (7/7). No changes that could be interpreted as pathology (perpendicular vessels, leukoplakia, etc.) were observed 3 months after radiotherapy.

At 6 months after radiotherapy, the previous changes in IPCLs were more evident in all patients, and the first changes in microarchitecture were observed. The most common change was ectasia of the IPCLs (5/7 patients). Another observed change was an increased number of vessels (3/7 patients).

**Fig. 1:**
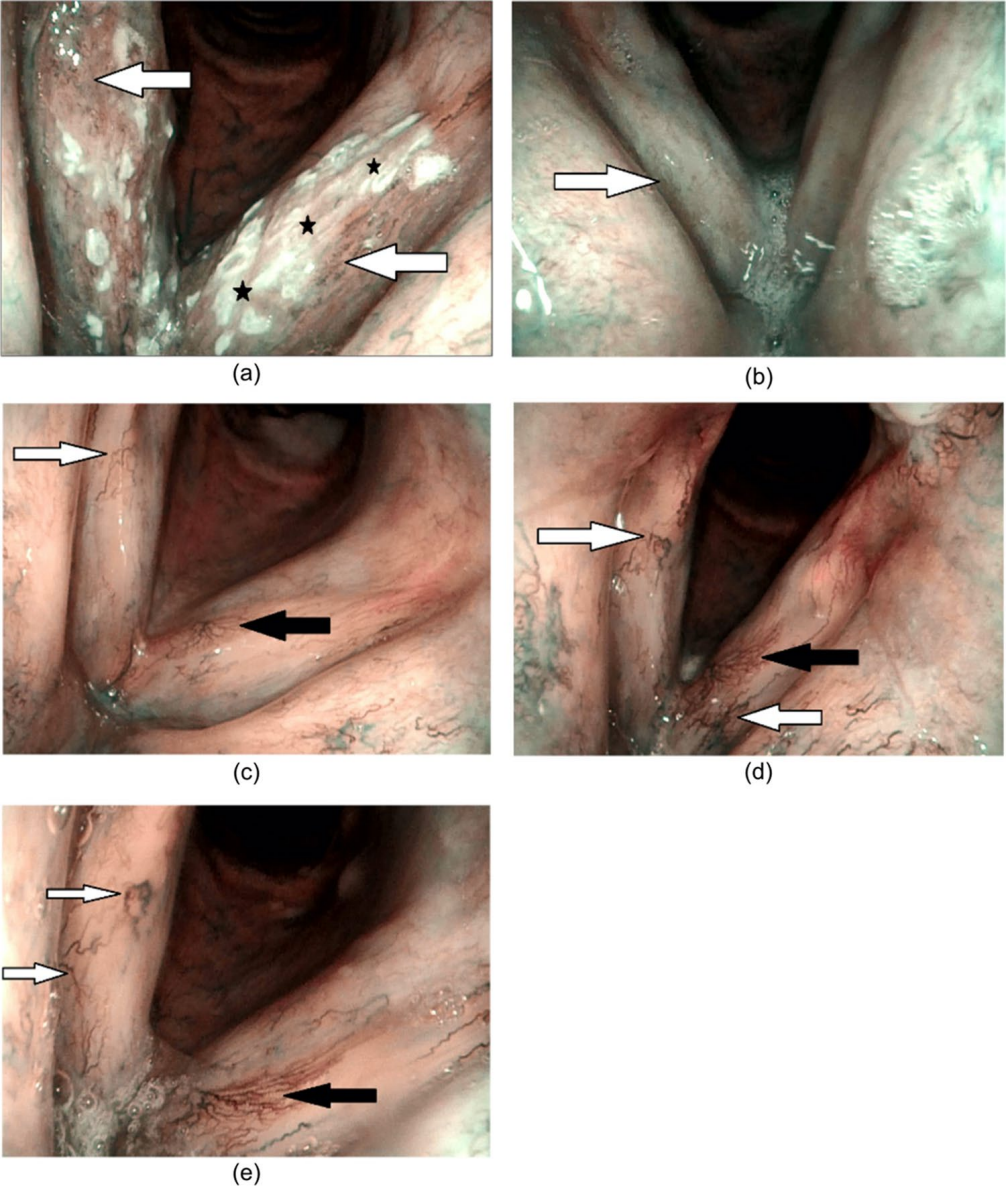
Changes in vocal cord mucosa before and after the radiotherapy (patient no. 3). **a** Before radiotherapy: perpendicular vasculature (white arrow) and leukoplakia (black star); **b** 3 months after radiotherapy: discrete, thin, hair-like, longitudinal vasculature (white arrow); **c** 6 months after radiotherapy: more pronounced longitudinal vasculature (white arrow); arborization of longitudinal vasculature (black arrow); **d** 9 months after radiotherapy: even more noticeable longitudinal vasculature, ectatic vessel (white arrow); pronounced arborization of longitudinal vasculature (black arrow); **e** 12 months after radiotherapy: longitudinal and ectatic vessels (white arrow), little to no progression; arborization of longitudinal vasculature (black arrow), no progression

**Fig. 2:**
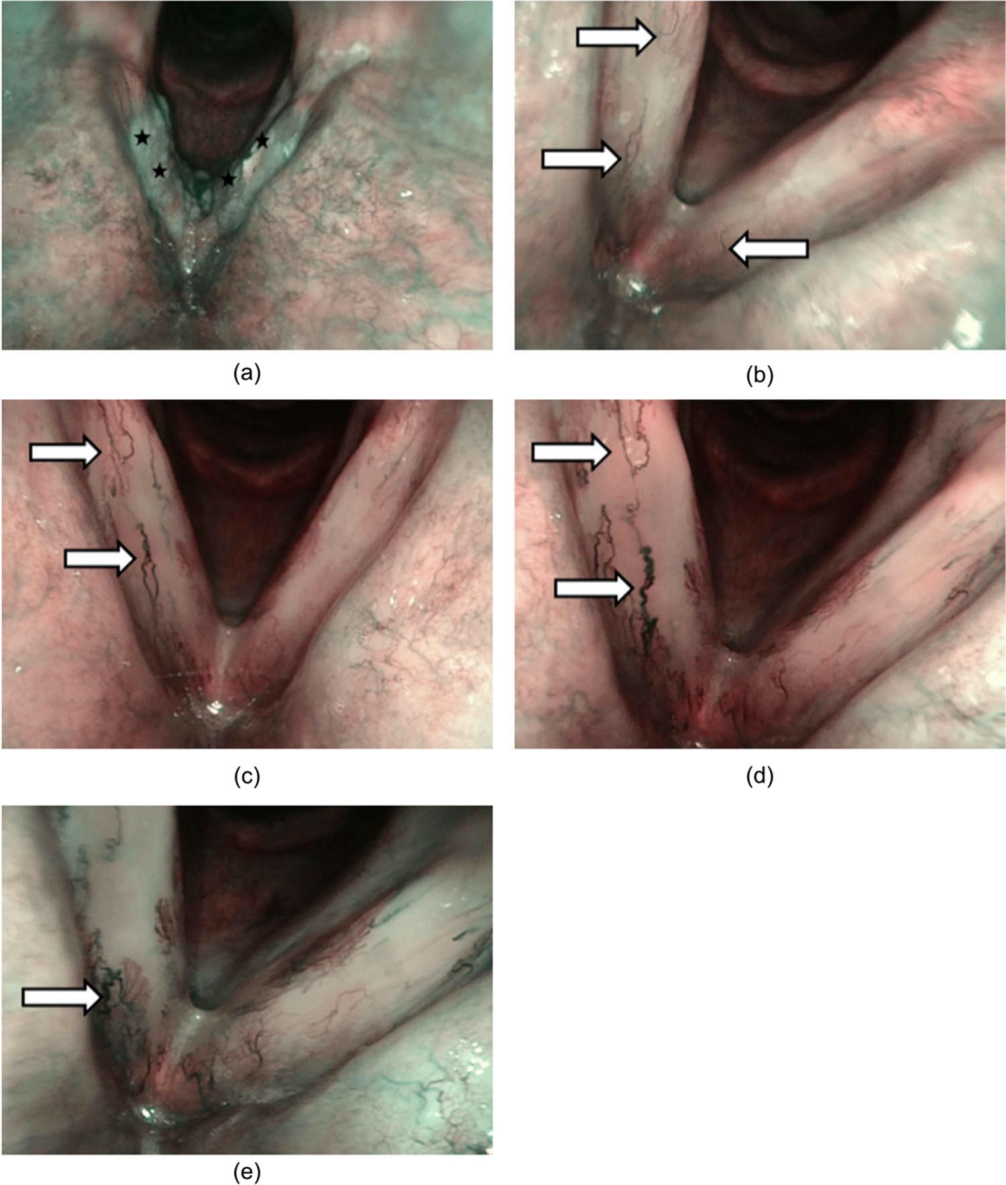
Changes in vocal cord mucosa before and after the radiotherapy (patient no. 5). **a** Before radiotherapy: leukoplakia (black star); **b** 3 months after radiotherapy: discrete, thin, hair-like, longitudinal vasculature (white arrow); **c** 6 months after radiotherapy: longitudinal ectatic vasculature (white arrow); **d** 9 months after radiotherapy: more noticeable longitudinal ectatic vasculature (white arrow); **e** 12 months after radiotherapy: longitudinal ectatic vasculature (white arrow), little to no progression

At the next follow-up, 9 months after radiotherapy, IPCLs were even more evident and distinctive. Longitudinal vasculature was still observed in all patients (7/7), and ectasia of the vessels was also observed in all of the patients (7/7). An increased number of IPCLs was reported in 4/7 patients. After the follow-up at 9 months, the progression of changes slowed down remarkably. At the 12-month follow-up, the progression of vascular changes was minimal in 4/7 patients, and there was no further progression of changes in IPCLs or their microarchitecture in 3/7 patients. A summary of the observed changes is given in Table 2, and examples of observed changes are given in Figs. 1 and 2.

**Table 2:** Overview of vasculature changes in the patients

Before RT	3 months after RT	6 months after RT	9 months after RT	12 months after RT
Leukoplakia, perpendicular vasculature	Discrete, thin, hair-like longitudinal vasculature	Previous changes more noticeable, first microarchitecture changes, ectatic vasculature	Previous changes even more noticeable, progression of microarchitecture changes	Minimal to no progression of previous changes

*RT* radiotherapy

## Discussion

The limitations of NBI technology have been widely discussed. First, the method requires clear visualization of the mucosa. Thus, if the mucosa is obscured by saliva, blood, or a layer of mucus, the examination is not useful [8]. In addition, a thick layer of hyperkeratosis may be found on some lesions. This phenomenon is called the umbrella effect and may occur in some lesions, particularly in leukoplakia or verrucous carcinoma [8]. This hyperkeratotic layer covers the IPCLs, and the pattern of the vasculature can no longer be seen [8]. Although the interpretation of such findings is very difficult, according to Stanikova et al., the vasculature surrounding the lesion can be used as an excellent diagnostic predictor of the type of lesion [9]. The second limitation is submucosal tumor growth, since NBI can identify only mucosal lesions. When a tumor grows submucosally, the shape of the IPCLs may look normal and the examination might have a false-negative result. The third major limitation mentioned by many authors is the influence of radiotherapy on IPCLs [3–5]. If the patient was treated by primary radiotherapy or by the combination of surgery followed by adjuvant radiotherapy, the interpretation of mucosal findings is difficult. The difficulties arise mainly from the fact that radiotherapy alters the characteristics of the laryngeal mucosa and induces inflammatory changes that affect the pattern of IPCLs [10]. Due to the influence of chronic inflammation on the vascular system, detection of possible tumor recurrence can be difficult and may not be diagnosed until an advanced stage, worsening the patient's prognosis [11].

In our study, pathologic changes in the vocal cord mucosa that were present before radiotherapy disappeared after radiotherapy. In the first 3 months after radiotherapy, the mucosal changes regressed and no changes previously observed and interpreted as pathological (perpendicular vessels, leukoplakia) were seen. The first discrete proliferation of new IPCLs was observed 3 months after radiotherapy. The new vessels were always longitudinal. Between 3 and 9 months, the IPCLs underwent significant changes. During this period, the IPCLs became much more visible and an increased number of vessels was observed. The microarchitecture of the vessels also changed during this period. The most common change was ectasia of the vessels. All changes during this period were longitudinal; no changes that could be interpreted as pathology were observed. After 9 months, the vascular changes became stable. Progression of mucosal changes between 9 and 12 months was minimal to nonexistent.

Accordingly, we propose three stages of IPCL changes after radiotherapy: the regression stage, the proliferation stage, and the stagnation stage.

The regression stage starts at the end of radiotherapy and lasts up to 3 months. The proliferation stage describes the main changes and mucosal vessels from 3 to 9 months. The stagnation stage describes the stagnation and de-escalation of changes in the IPCLs seen at 9 months after radiotherapy.

The reason for the appearance of longitudinal vessels after radiotherapy is probably that radiation tends to cause chronic inflammation in tissues [10]. Many classification systems (such as the Ni, ELS, or the Puxxedu classification system) tend to interpret longitudinal vascular changes as benign, and some of the classifications even state that certain types of vascular changes (Ni type II–III, Puxxedu type I) are specific to inflammation [7, 12]. This is also supported by the fact that inflammatory lesions of the vocal cords (such as Reinke edema or chronic laryngitis) tend to have very similar longitudinal vascular changes, although the microarchitecture tends to be different. The caliber of the vasculature in inflammatory lesions tends to be homogeneous, without arborization, whereas the caliber of vessels that change after radiation therapy is usually not homogeneous, and arborization may be present.

It must be said that the study was limited to a small sample of patients. The number of patients was limited, mainly because the preferred treatment modality for T1 vocal cord carcinoma is cordectomy. This treatment provides good patient outcomes and was the main reason patients were excluded from the study [13]. Although a statistically small sample was used, the consistency of the vascular changes observed was very high and the changes were very similar in all patients in the study.

After the radiotherapy, no perpendicular vasculature was recorded. Therefore, we propose that no special follow-up procedures are required and that patients should be followed in accordance with standard protocols proposed by the European Laryngological Society for patients after treatment for laryngeal cancer [14]. If the patient was treated with curative radiotherapy for glottic carcinoma and the radiation dose was 55 Gy, the longitudinal vessels should be interpreted as radiation-induced inflammatory changes. On the other hand, if perpendicular vasculature is present, the situation should be managed with caution due to probable tumor recurrence, and a sample should be taken for histological examination.

## Conclusions

IPCL changes induced by radiation therapy with a dose of 55 Gy for glottic cancer are only longitudinal. Any appearance of perpendicular vasculature after curative radiotherapy must raise suspicion of malignant tumor recurrence and should be thoroughly examined under general anesthesia.
